# Zn-Based Deep Eutectic Solvent as the Stabilizing Electrolyte for Zn Metal Anode in Rechargeable Aqueous Batteries

**DOI:** 10.3389/fchem.2021.825807

**Published:** 2022-01-14

**Authors:** Gaurav M. Thorat, Van-Chuong Ho, Junyoung Mun

**Affiliations:** Department of Energy and Chemical Engineering, Incheon National University, Incheon, South Korea

**Keywords:** Zn anode, deep eutectic solvent, electrolyte, zinc ion batteries (ZIBs), des

## Abstract

Owing to its low cost and high safety, metallic zinc has received considerable attention as an anode material for zinc aqueous batteries (ZIBs). However, the Zn metal instability as a result ultrafast of obstinate dendrite formation, free-water-induced parasite reactions, and corrosive electrolytes has detrimental effects on the implementation of ZIBs. We present an alternative stable electrolyte for ZIBs based on a zinc chloride/ethylene glycol deep eutectic solvent (DES). This electrolyte consists of abundant low-cost materials and a utilizable Zn^2+^ concentration of approximately 1 M. It combines the advantages of the aqueous and DES media to provide safe and reversible Zn plating/stripping with a two-fold increase in the cycling life compared to that of conventional aqueous electrolytes. With these advantages, the Zn symmetric cell operates at 0.2 mA cm^−2^ for 300 h. Due to its high efficiency and compositional versatility, this electrolyte enables the investigation of a non-aqueous electrolyte family for ZIBs that fulfill grid-scale electrical energy storage requirements.

## Introduction

Zinc-ion batteries (ZIBs) have attracted worldwide interest for their application in next-generation energy storage devices due to their notable characteristics, such as cost effectiveness, eco-friendliness, high safety, resource abundance, high volumetric capacity, and aqueous electrolyte compatibility (Hu et al., 2020; Wang et al., 2020a; Hansen and Liu, 2021). The electrolytes in ZIBs are essential components that play a critical role in their electrochemical behavior (Zhang et al., 2020). They provide a steady electrochemical potential window, channel between the anode and the cathode for Zn movement, and aid in determining the reaction mechanism and the ionic conductivity (IC) (Zhu et al., 2021). ZIBs with aqueous electrolytes possess several benefits, such as high energy density (Wu et al., 2021b), safety, and stability over a high number of cycles, as well as ecological benignity (Zhang et al., 2018b; Mo et al., 2019; Zhu et al., 2019). However, accompanying these advantages are some difficulties as well. During electrochemical cycling in alkaline electrolytes, significant amounts of dendritic growth and/or by-products, such as ZnO and Zn(OH)_2_, accumulate on the Zn surface, preventing further application due to low Coulombic efficiencies (CEs) and rapid capacity fading. In neutral or mildly acidic aqueous electrolytes, the Zn anode is primarily concerned with hydrogen evolution, electrolyte breakdown due to the small potential window of water (1.23 V), and Zn electrode passivation, all of which affect the battery efficiency (Zhang et al., 2020; Liu et al., 2021). The use of water-in-salt (WIS) electrolytes, such as highly concentrated Zn-TFSI, ZnCl_2_, LiTFSI/Zn[N(SO_2_CF_3_)_2_]_2_, LiN(SO_2_CF_3_)_2_, and LiN(SO_2_CF_3_)_2_/CF_3_SO_3_Li, offers an excellent strategy for engineering a high-stability Zn anode by forming a solvation sheath structure of the Zn^2+^ cation, which eliminates any water-induced side reactions (Zhang et al., 2018a; Zhao et al., 2019; Kao-ian et al., 2021). Another advantage of WIS electrolytes over dilute electrolytes is the thermodynamically shifted potential equilibrium of the cation-intercalation reaction. However, these WIS solutions contain a high proportion of fluorinated salts, which limits their practical viability owing to their high cost and toxicity. Another inorganic additive MXene nanosheets in the electrolyte significantly decreased Zn^2+^ diffusion paths and aided migration, and the numerous functional groups and high conductivity caused uniform nucleation (Du et al., 2021; Sun et al., 2021). In comparison to organic and inorganic additives, the organic polymer additives effectively control electrodeposition rate however their synthesis is tedious (Du et al., 2021; Wu et al., 2021a). Therefore, these issues associated with electrolytes must be addressed before ZIBs can be effectively used on a large scale.

Another potentially viable electrolyte is a room-temperature ionic liquid (RTIL) electrolyte, which exhibits several desirable properties, such as a high IC, good dissolving ability, wide potential window, low volatility, and nonflammability. For example, dendrite-free plating/stripping (>1,500 h) and 100% CE were achieved using an IL-based Zn salt electrolyte. Traditional RTILs, imidazolium-based ionic liquids, exhibit an excellent Zn electrodeposition performance as well as an exceptional electrochemical window (>4 V) (Kazemiabnavi et al., 2016). However, RTILs have proven to be expensive and extremely susceptible to moisture, making large-scale production of ZIBs using RTIL impossible (Abbott et al., 2008; Abbott et al., 2013). In this context, a promising and innovative ionic fluid, a deep eutectic solvent (DES) analogous to an ionic liquid, is rapidly emerging in the field of Li-ion battery electrolytes. DESs are formed by mixing two or more components in proper molar ratios simultaneously, resulting in a eutectic mixture via hydrogen bonding. Due to their high viscosity, conductivity, surface tension, and biodegradability, DESs are considered as green solvents (Thorat et al., 2018).

Herein, we report the development of a cost-effective, environmentally friendly, and intrinsically safe ZnCl_2_–4 ethylene glycol (EG) deep eutectic composition (ZnCl_2_–4EG DES) as an electrolyte for Zn anodes in ZIBs. The properties of EG, which acts as a water blocker to generate a confined high-concentration electrolyte (Wang et al., 2020b; Xu and Jiang, 2021), and ZnCl_2_, which was previously used as a WIS electrolyte for a dendrite-free Zn metal anode, make them ideal components for DESs used as electrolytes for ZIBs. Furthermore, early reports indicate that ZnCl_2_-based DESs have potential applications in electrochemistry. In particular, the ZnCl_2_–4EG DES has been previously used in electrochemical processes (Abbott et al., 2007a). When tested for the Zn symmetric cell, this electrolyte combines the advantages of both aqueous and DES media to enable safe and reversible Zn plating/stripping with a two-fold increase in the cycling life compared to that of conventional aqueous electrolytes.

## Experimental Section

### Materials

Ethylene glycol (EG) (HOCH_2_CH_2_OH, > 99.5%, hydrogen bond donor (HBD)) and Zinc chloride (ZnCl_2_ > 99.9%, hydrogen bond acceptor (HBA)) were procured from Sigma Aldrich (South Korea) and used without further purification. Zn foil (99.995%, Alfa Aesar, South Korea) with a thickness of 0.025 cm was used.

### Deep Eutectic Solvent Electrolyte Preparation

DESs were formed by mixing ZnCl_2_ and EG in a specified molar ratio (1:4) and heating at 100°C for 1 h at air pressure under steady magnetic stirring until a homogeneous liquid was obtained. In subsequent discussion, the synthesized electrolyte will be referred to as the ZE DES. Finally, the synthesized DES solution was sealed and placed in a transparent glass bottle for later use. The decrease in the T_m_ of the mixture is caused by charge delocalization due to hydrogen bonding between the halide anion and EG.

### Characterization of Materials

Fourier transform infrared spectra were recorded on a PerkinElmer Spectrum Two ATR instrument over a range of 4,000–400 cm^−1^. The thermal stability of the ZE electrolyte was analyzed using thermogravimetric analysis (TGA). TGA was performed on a Scinco TGA-N 1500 instrument over a range of 30–800°C under nitrogen flow at a scanning rate of 10°C min^−1^. Differential scanning calorimetry (DSC) was carried out using PerkinElmer DSC 4000.

### Assembly of Symmetric Cells

Two bare Zn foil electrodes with a diameter of 12 mm each were assembled into CR2032-type coin cells. A ZnCl_2_–4EG DES was used as the electrolyte, and glass fiber filters served as separators. The cells were then sealed in air.

### Electrochemical Tests

To explore the electrochemical behavior of Zn foils, galvanostatic charging-discharging cycling was performed at a current density and total capacity of 0.2–2 and 0.5–2 mAh cm^−2^, respectively. The cycling performance was evaluated using a standard eight-channel WonA-Tech battery test system (South Korea). Cyclic voltammetry (CV) was conducted to evaluate the electrochemical behavior of the Zn anode in an electrode cell with glassy C, Pt wire, and Ag wire serving as the working, counter, and reference electrodes, respectively. A ZE DES was used as the electrolyte.

## Results and Discussion

**FIGURE 1:**
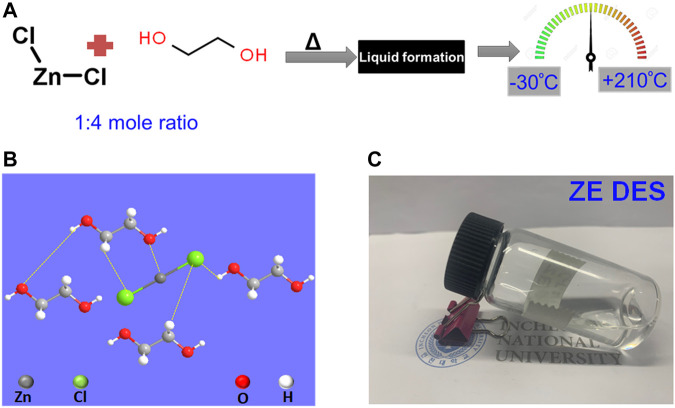
**(A)** Synthesis scheme for the preparation of the ZnCl_2_–4EG DES electrolyte. **(B,C)** Schematic and photographs of the ZE DES electrolyte.

First, the ZnCl_2_-based eutectic solvent used was prepared by simply mixing ZnCl_2_ with EG in a 1:4 mole ratio and stirring at 100°C until a transparent liquid, named as ZE DES, was formed. The preparation methodology for the ZE DES is shown in Figure 1A. The molar fraction between ZnCl_2_ and EG was chosen to be 1:4, as it forms the most stable structure relative to other molar fractions via the formation of a special molecular packing (Tran and Thi Hang, 2018; Kalhor et al., 2021). The larger clusters of EG disintegrate into smaller ones upon mixing with ZnCl_2_, and ZnCl_2_–EG complexes are formed. Theoretical calculations indicated that EG molecules interacted with ZnCl_2_ via the production of both O–H–Cl hydrogen bonds and Zn–O coordination bonds (Figure 1B). ZnCl_2_ is a linear molecule with a 180° Cl–Zn–Cl angle. Upon mixing with EG, the hydroxyl groups of EG engage with the Zn metal atoms, thereby decreasing the Cl–Zn–Cl angles. As a result, the Zn–Cl bond length increases with increasing EG concentration, which is noteworthy. This is due to the partly ionic character of ZnCl_2_, and therefore, the high EG content in the complexes causes a decrease in the electron density at the Zn–Cl bond critical point. As a result, the Zn–Cl bond tends to dissociate in the ZnCl_2_-based DES resulting in high electrical conductivity compared to that of EG (Sarjuna and Ilangeswaran, 2020; Kalhor et al., 2021). The ZE DES used in this study has a freezing point lower than room temperature, making it suitable for a variety of room-temperature applications. In general, the natures and molar compositions of the metal salt (ZnCl_2_) and the HBD (i.e., EG) affect the physical and chemical features of deep eutectics. The formed ZE DES displayed a colorless, transparent, and uniform state without any additional aqueous solution (Figure 1C).

**FIGURE 2:**
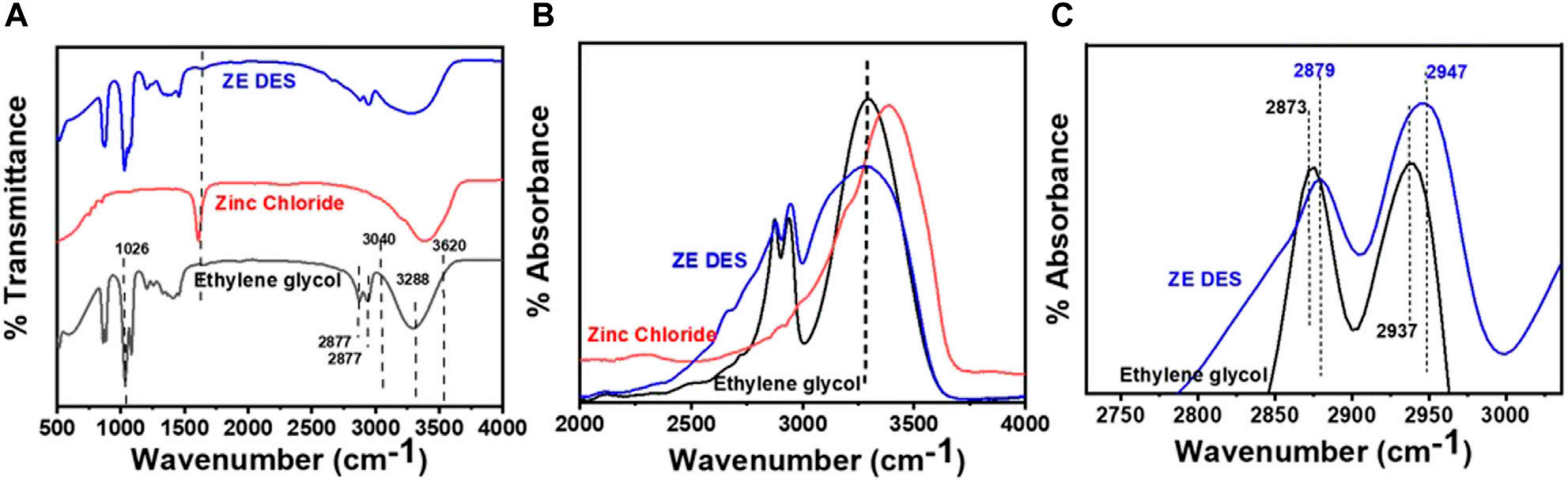
**(A)** Fourier transform infrared spectra of neat ethylene glycol, ZnCl_2_, and their eutectic mixture (ZE DES). **(B)** −OH and **(C)** -methylene stretching regions.

As previously mentioned, the ZE DES exhibited a diverse range of interactions, including hydrogen bonding, electrostatic interactions, and dispersion. Among these, hydrogen bonding interactions have received significant attention. Owing to the exceptional sensitivity of IR to hydrogen bonding interactions, FT-IR spectra were acquired to investigate the chemical bond structures in the DESs. Figure 2A shows the infrared absorption spectra of the ZE DES, pure EG, and ZnCl_2_. The total infrared spectrum of ZE DES is identical to that of ZnCl_2_ and EG, but with a frequency shift in the *v*(O–H) and (C–H) regions. These findings indicate that the EG structure was not destroyed during the DES formation process. For pure EG, the broad band centered at 3,288 cm^−1^ in the *v*(O–H) region extending from 3,040 to 3,620 cm^−1^ is strongly related to the extensive hydrogen bonding network. The two bands in the (C–H) region at 2,860 and 2,941 cm^−1^ are attributed to the s(C–H) and as(C–H) of methylene, respectively. The peaks at 1,026 cm^−1^ can be attributed to the stretching vibrations of the C−C−O bonds (Hong et al., 2016; Kalhor et al., 2021). When ZnCl_2_ was added, the band shapes, positions, and intensities changed significantly.

The strong Peak in ZnCl_2_ at 1,608 cm^−1^ is assigned to ν (H-OH) which may originated from the absorption of water by higley hygroscopic zinc chloride during FTIR measurement. Its position depends on hydration number of ZnCl_2_. The presence of such prominent peak has been also confirmed in the study of crystalline and liquid structure hydration number (R) (Wilcox et al., 2015). However, DES synthesis is carried out at higher temperature and subsequent storage is strictly controlled where exposure the water to DES is minimum. As result the Peak at 1,608 cm^−1^ resulted into diminishing the intensity with blue shift at 1,647 cm^−1^ (reduction in hydration number in ZnCl_2_) upon DESs formation which can clearly see from the enlarge portion of corresponding region of IR depicted in Supplementary Figure S1 (Electronic supplementary information; ESI).

In the ν(O–H) region, the -O-H stretching peak shifts to a wavenumber lower than 3,288 cm^−1^ (Figure 2B). Furthermore, the stretching (C–H) and asymmetric stretching (C–H) bands are blue-shifted by 5 and 10 cm^−1^, respectively (Figure 2C). These results show that ZnCl_2_ has a significant impact on the hydrogen bonding characteristics of EG. These blue and red shifts of the IR bands are typically linked to the strength variations of the respective hydrogen bonds in the corresponding molecules. A red shift in the band indicates stronger hydrogen bonds, while a blue shift indicates weaker hydrogen bonds for appropriate hydrogen bonding (McDowell, 2003; Sanchora et al., 2019). However, for an improper hydrogen bond forming group, the band shifts were in the reverse direction. Since the C–Hs of EG are inappropriate hydrogen bond-formation groups, the blue shifts represent stronger hydrogen bonds in ZE DES than those in pure EG.

**FIGURE 3:**
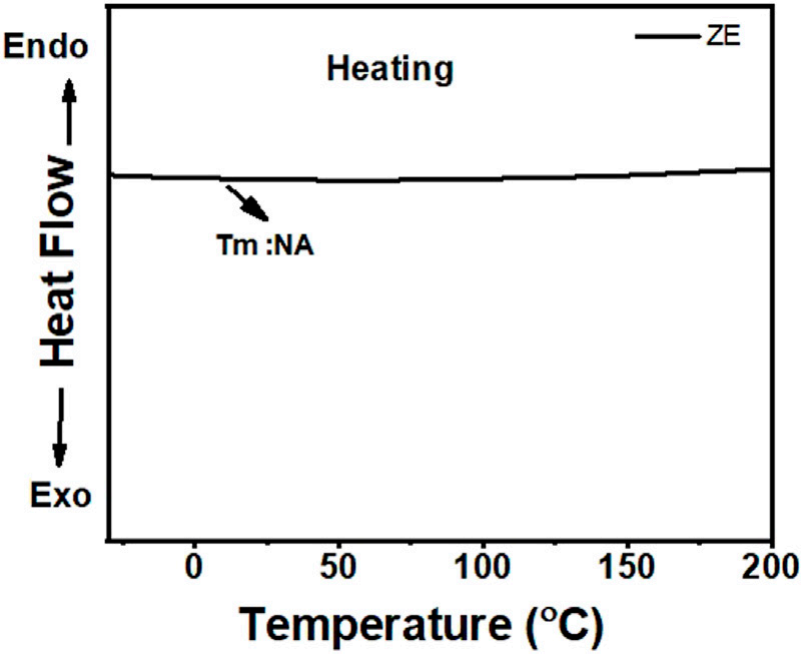
DSC traces of the ZE DES electrolyte at −30 to 200°C with a heating rate of 5°C min^−1^.

TGA and DSC measurements of the ZE DES were conducted to investigate the thermal characteristics of the electrolyte under study. The DSC measurement results of the ZE DES electrolyte are shown in Figure 3. In the ZE DES electrolyte, there was no discernible peak corresponding to the melting point of EG (−12°C) (Kaur et al., 2018), indicating that deep eutectics were formed in the absence of unreacted starting components. Therefore, the quantitative generation of deep eutectics through the combination of metal salt and alcohol, for example, EG, could be crucial for lowering the melting temperature.

**FIGURE 4:**
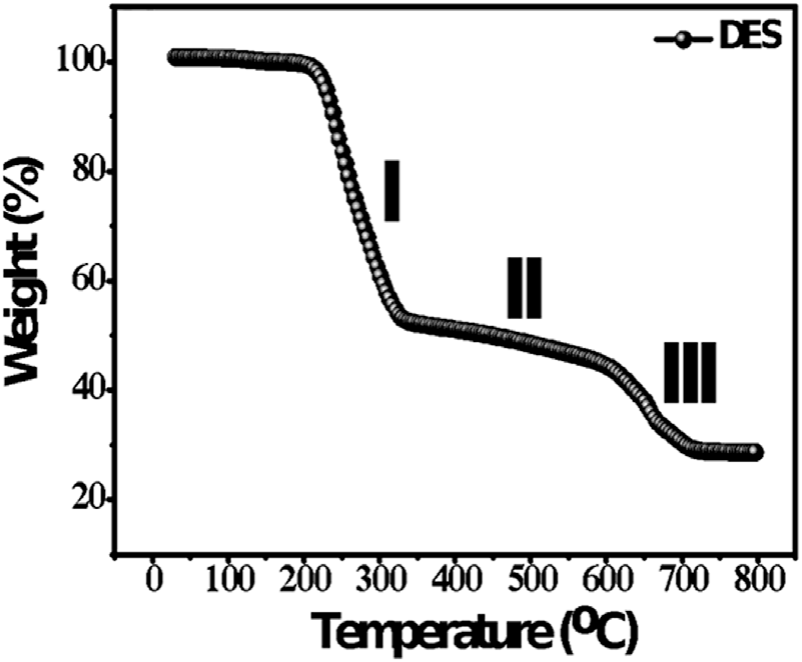
TGA analysis of the ZE electrolyte in N_2_ atmosphere at a heating rate of 5°C min^−1^.

Figure 4 shows the TGA results of ZE DES. The electrolyte exhibits the greatest weight loss between 200 and 400°C, making it suitable for high-temperature applications. At temperatures close to the boiling point of pure organic components, the Zn eutectic solvent loses its organic component rather than decomposing (Wang et al., 2018). The DSC and TGA results together suggest that the ZE electrolyte is stable over a wide temperature range and should be superior to most aqueous and aprotic electrolytes used for ZIBs.

### Electrochemical Analysis

The electrochemical properties of the Zn eutectic electrolyte were investigated to ascertain its applicability in actual batteries. Initially, the electrochemical stability of the Zn eutectic mixture was investigated using a conventional three-electrode configuration comprising glassy C, Pt wire, and Ag wire immersed in a ZE electrolyte as the working, counter, and reference electrodes, respectively.

**FIGURE 5:**
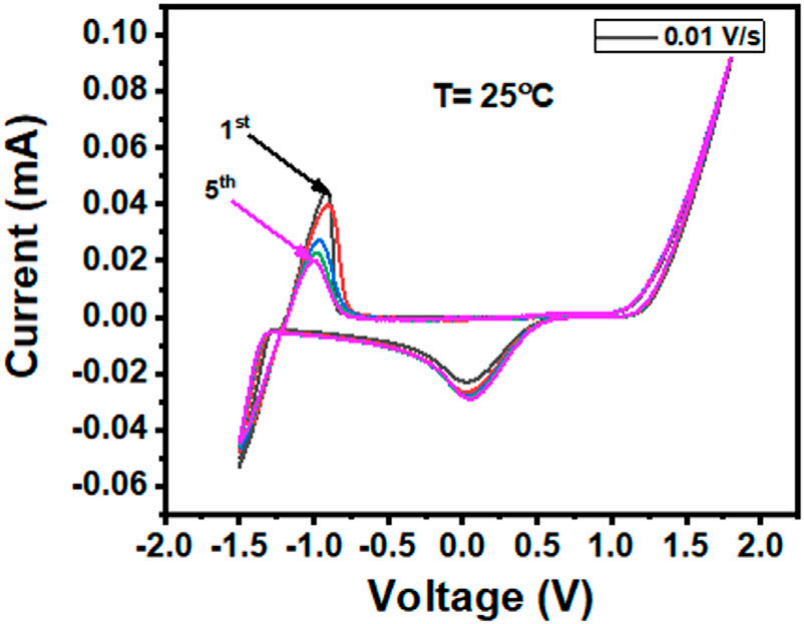
Cyclic voltammogram profile recorded at a sweeping rate of 0.01 V s^−1^ showing the electrochemical window of the Zn electrolyte.

The cyclic voltammogram profile of the Zn eutectic combination (Figure 5) revealed that potentials less than −1.2 V resulted in Zn^2+^ electroplating, while those greater than 1.05 V vs. Ag wire resulted in EG breakdown. Therefore, the liquid has an impressive potential window of 2 V, which is limited by Zn electroplating at the cathodic end and gas evolution at the anodic end of the liquid. Similar to that of the choline chloride/2ZnCl_2_ type 1 DES system, there is some indication of under-potential deposition at 0.05 V (Abbott et al., 2007b). Despite the Zn mole fraction and Zn species being significantly different in the ZE electrolyte (Type IV DES system), the potential window is nearly identical to those of their type I counterparts, which are widely used in electrochemical processes.

**FIGURE 6:**
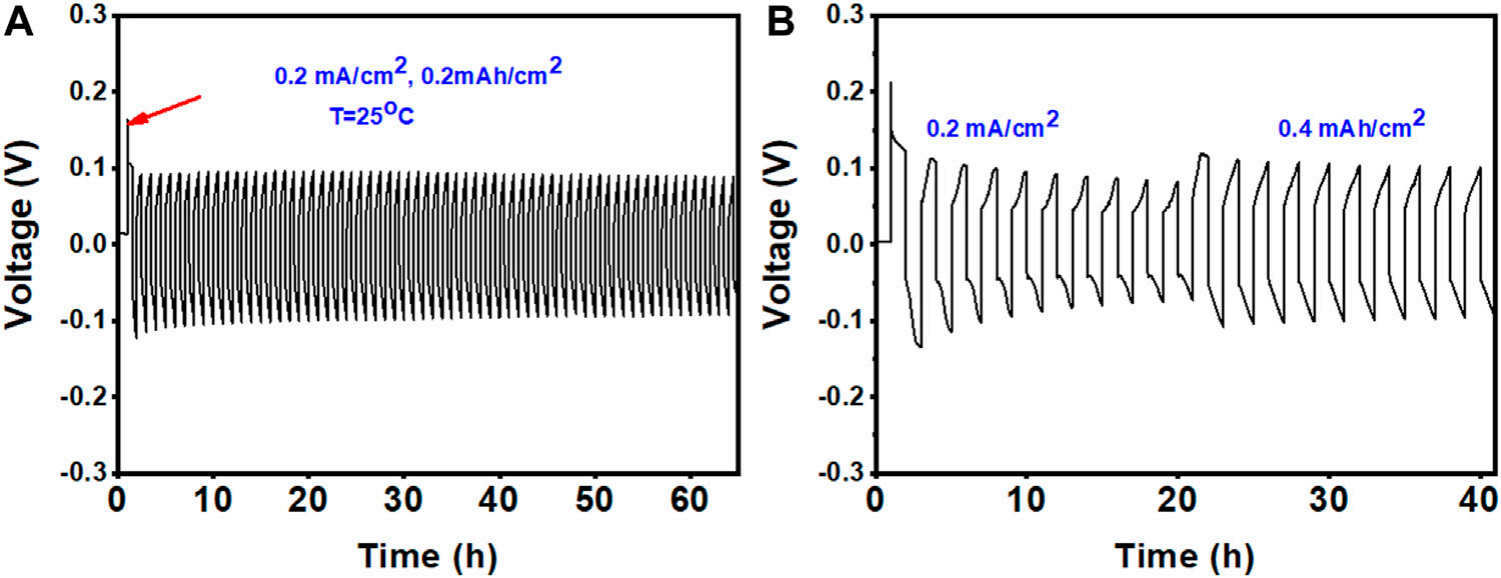
Stripping/plating performance of the Zn metal anode in the ZE electrolyte at different current densities: **(A)** 0.2 mAh cm^−2^ and **(B)** first five cycles at 0.2 and 0.4 mAh cm^−2^.

Another important aspect of Zn electrodes for ZIBs is the reversible plating/stripping of Zn metal anodes. The reversibility and stability of Zn in the ZE DES electrolyte were investigated using a Zn/Zn symmetric cell, where Zn electrodes were used as both positive and negative electrodes under galvanostatic conditions. Figures 6A,B show the results of cycling experiments in Zn|electrolyte|Zn cells at different current densities in the ZE electrolyte. During cycling, the polarization voltage was constrained by the maximum and minimum voltages at each current density.

**FIGURE 7:**
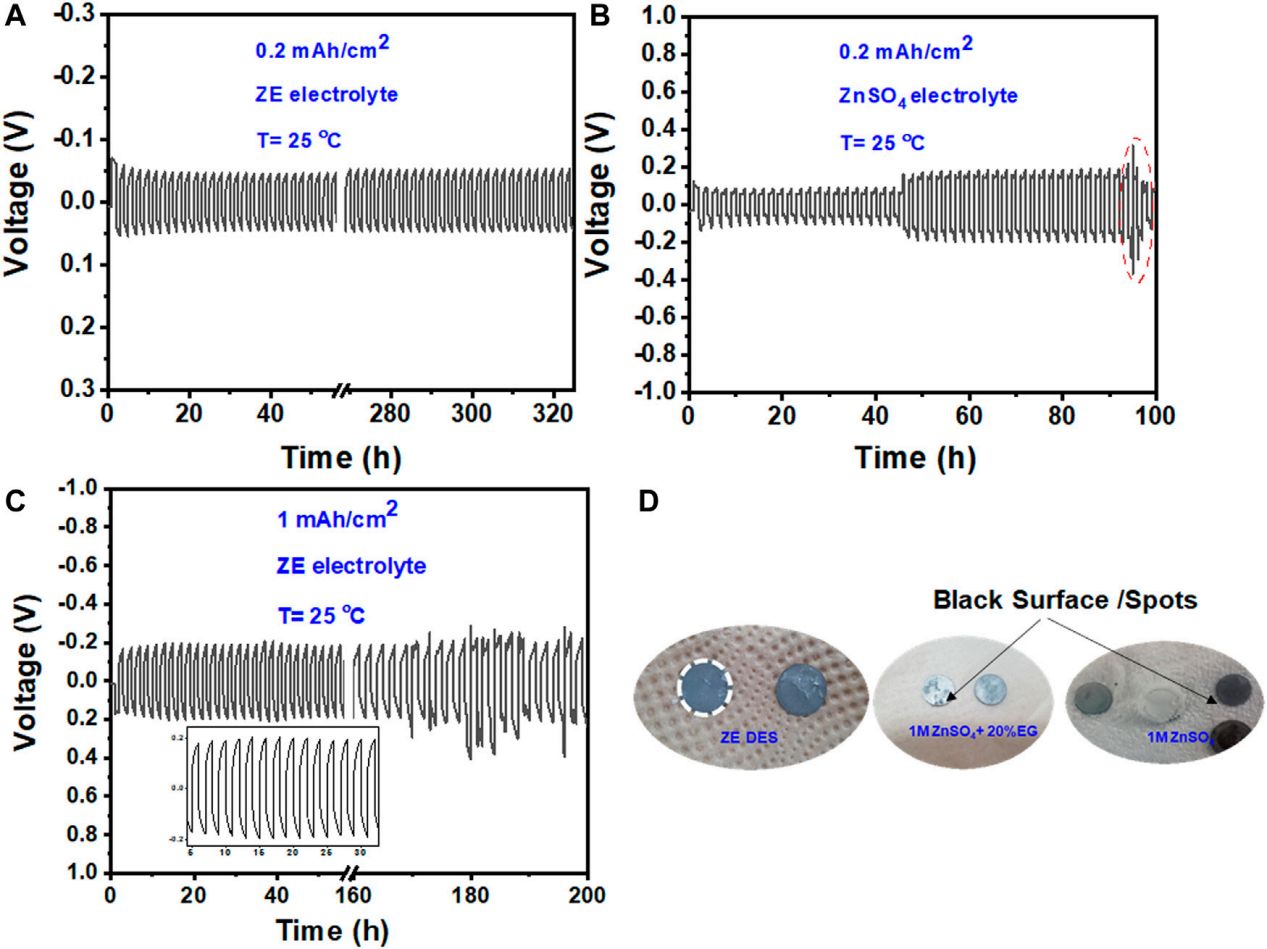
Long-term stripping/plating performance of the Zn metal anode at 0.2 mAh cm^−2^ in **(A)** ZE electrolyte and **(B)** aqueous 1 M ZnSO_4_, and **(C)** at the 1 mAh cm^−2^ for ZE electrolyte. **(D)** Digital photographs of the Zn foil acquired after 50 cycles at 0.2 mAh cm^−2^.

Figure 6A shows that a relatively substantial initial overpotential is required to trigger the Zn plating/stripping reaction (shown by the red arrow), but the overpotential is reduced during consecutive cycles. Furthermore, the maximum voltages did not increase during cycling, demonstrating that no passivation layer was formed on the Zn surface (Cui et al., 2021). The excellent performance of the Zn metal anode was further validated by long-term cycling at different current densities, as shown in Figure 7A. The symmetric cell operates steadily over 180 cycles (>300 h) at 0.25 mAh cm^−2^, indicating the high reversibility of the Zn anode. The polarization curve remained stable throughout the entire cycle process. On the other hand, a gradual increase in overpotential values was observed for symmetric cells (Zn/Zn) cycled at 0.25 mAh cm^−2^ in the conventional aqueous ZnSO_4_ electrolyte. Zn plating/stripping could only be cycled for 90 h at 0.25 mAh cm^−2^ in the aqueous ZnSO_4_ electrolyte (Figure 7B).

After 50 cycles at 0.2 mAh cm^−2^, we opened the Zn/Zn cell with both the aqueous ZnSO_4_ and ZnSO_4_/EG/H_2_O electrolytes and found that the Zn foil possessed some black spots that would further grow and lead to cell death (Figure 7D). In contrast, the cell-cycled ZE electrolyte displayed no such black spots, indicating the high stability of the Zn anode in the ZE electrolyte. Benefiting from these features, the Zn/Zn symmetric cell showed excellent stability for 200 h at a high current density of 1 mAh cm^−2^ with an overall overpotential value of less than 200 mV (Figure 7C).

**FIGURE 8:**
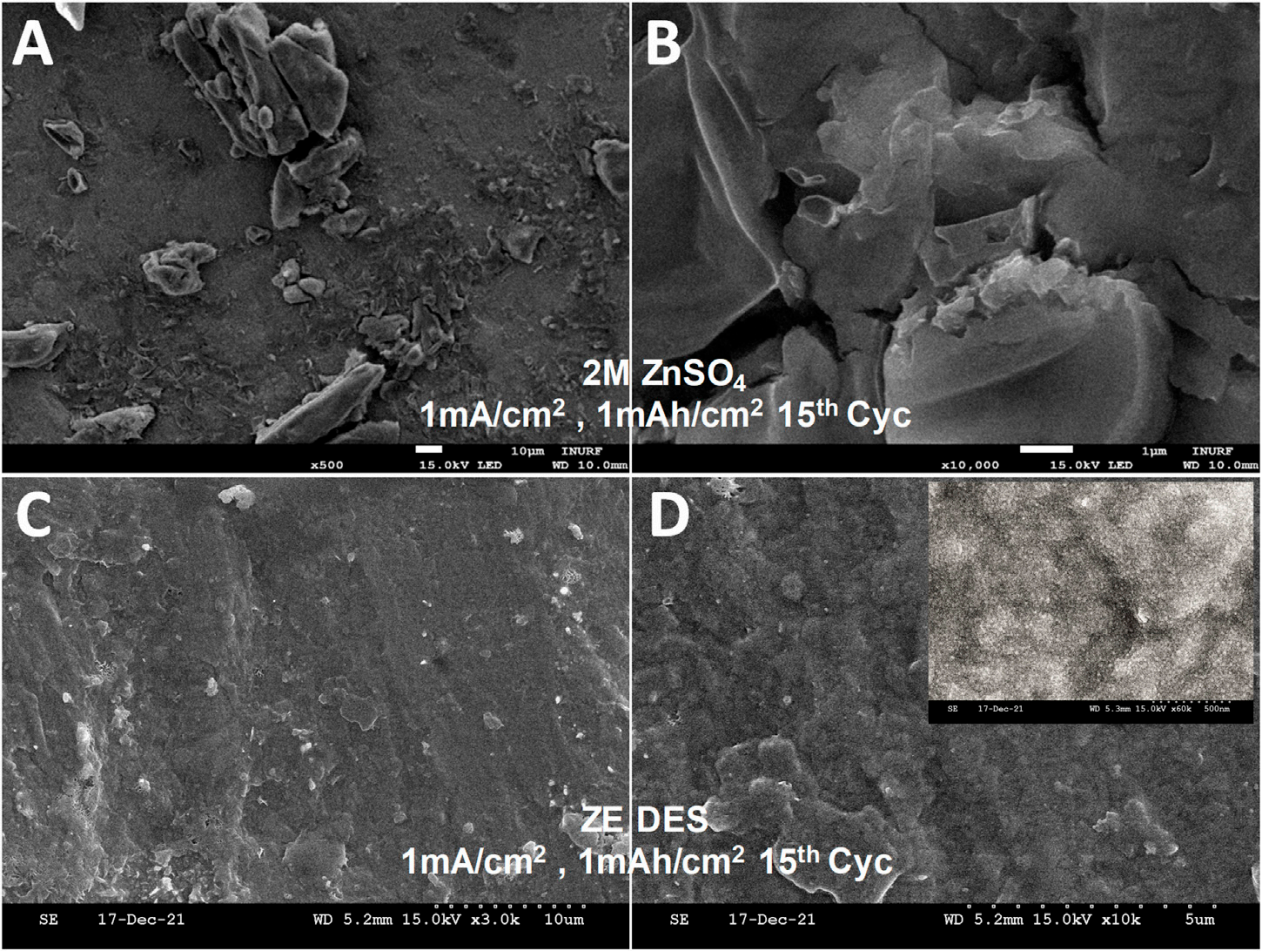
SEM images of Zn metal anode **(A,B)** 2 M ZnSO_4_ electrolyte ZE electrolyte; **(C,D)** ZE electrolyte after 15 cycles at 1 mAh cm^−2^.

Figure 8 shows SEM images of the typical morphology of Zn metal growths deposited in ZE DES and ZnSO_4_ electrolyte at current density of 1 mA cm^−2^ with a total charge of 1 mAh cm^−2^. Significant difference in morphologies is observed. After the 15th cycle, several large Zn protrusions were found in the case of aqueous ZnSO_4_, but a smooth surface with relatively few large particles was observed in the case of Zn anode cycled in ZE electrolyte. As shown in high resolution SEM image, the Zn surface is mostly composed of small spherical particles (Inset of Figure 8D). The Zn symmetric cell further operates for 40 h at 2 mA cm^−2^ with capacity of 2 mAh cm^−2^ indicating good reversibility even at high current density (Supplementary Figure S2). Supplementary Table S1 summarizes the performance comparison of ZE DES electrolyte with other DESs and EG-based electrolytes. The excellent cycling performance demonstrates the electrolyte’s remarkable potential for ZIBs.

## Conclusion

A biocompatible, stable, and low-cost Zn-based deep eutectic solution was successfully fabricated in this study to stabilize the Zn metal anode in ZIBs with a larger stability window. Aqueous and DES media were combined in this electrolyte to allow for safe and reversible Zn plating and stripping, with a two-fold increase in the cycling life compared to that of traditional aqueous electrolytes. This electrolyte with improved Zn anode reversibility and cycling stability could lead to a higher energy density and more stable ZIBs than conventional aqueous ones.

## Data Availability

The original contributions presented in the study are included in the article/Supplementary Material, further inquiries can be directed to the corresponding author.
