# Supplementary material for: Zn-Based Deep Eutectic Solvent as the Stabilizing Electrolyte for Zn Metal Anode in Rechargeable Aqueous Batteries
Source: Front Chem. 2022 Jan 14;9:825807. doi: 10.3389/fchem.2021.825807 (PMC8795620; doi:10.3389/fchem.2021.825807)
Supplement: Supplementary file 1 [file DataSheet1.docx]

**Zn**-**based deep eutectic solvent as the stabilizing electrolyte for Zn** **metal anode in rechargeable aqueous batteries**

Gaurav M. Thorat^1^, Van-Chuong Ho^1^, and Junyoung Mun*^,1^

^1^Department of Energy and Chemical Engineering, Incheon National University, 12-1, Songdo-dong, Yeonsu-gu, Incheon 22012, Republic of Korea

**Corresponding Author**

*Email: [jymun@inu.ac.kr](mailto:jymun@inu.ac.kr), Telephone: +82-32-835-8876, Fax: +82-32+835-4866


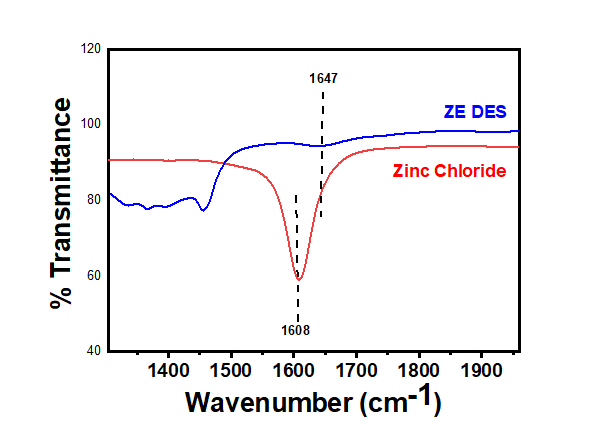


**Figure S1** Fourier transform infrared spectra of neat ZnCl_2_, and eutectic solvent (ZE DES).


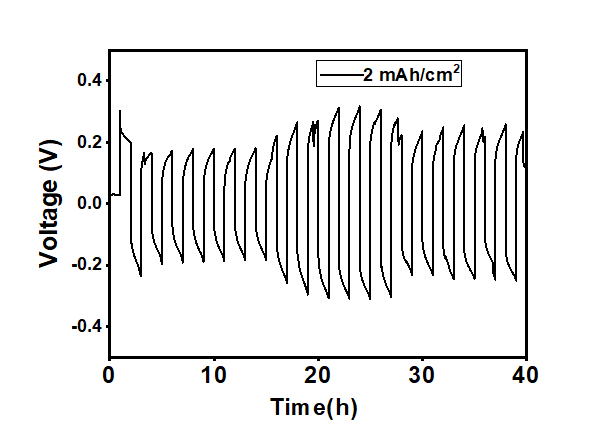


**Figure S2** Stripping/plating performance of the Zn metal anode in the ZE electrolyte at different current densities: 2 mAh cm^−2^; 2mAh cm^−2^.

**Table S1-** Performance comparison table for Zn symmetric cell in DES and ethylene glycol-based electrolytes

| **Electrolyte system** | **Current density (mA/cm^2^)** | **Stability (h)**  **(Symmetric cell)** | **Reference** |
| --- | --- | --- | --- |
| ZnCl_2_-acetamide (ZES-2) | 0.1 | 150 | (Shi et al., 2021) |
| ZnCl_2_-acetamide | 0.2 | 150 | (Wang et al., 2018) |
| ChCl/urea-0.3 MZnCl_2_ | 1 | 10 | (Kao-ian et al., 2019) |
|  | 0.5 | 10 | (Kao-ian et al., 2019) |
| ZnSO_4_-15% Ethylene glycol | 1 | 170 | (Wang et al., 2020) |
| ZnCl_2_-Ethylene glycol  (ZE DES) | 1 | 180 | Present work |
| ZnCl_2_ Ethylene glycol  (ZE DES) | 0.2 | 350 | Present work |

# **References**

Kao-ian, W., Pornprasertsuk, R., Thamyongkit, P., Maiyalagan, T., and Kheawhom, S. (2019). Rechargeable Zinc-Ion Battery Based on Choline Chloride-Urea Deep Eutectic Solvent. *Journal of The Electrochemical Society* 166, A1063–A1069. doi:10.1149/2.0641906JES/XML.

Shi, J., Sun, T., Bao, J., Zheng, S., Du, H., Li, L., et al. (2021). “Water-in-Deep Eutectic Solvent” Electrolytes for High-Performance Aqueous Zn-Ion Batteries. *Advanced Functional Materials* 31, 2102035. doi:10.1002/ADFM.202102035.

Wang, N., Yang, Y., Qiu, X., Dong, X., Wang, Y., and Xia, Y. (2020). Stabilized Rechargeable Aqueous Zinc Batteries Using Ethylene Glycol as Water Blocker. *ChemSusChem* 13, 5556–5564. doi:10.1002/CSSC.202001750.

Wang, Y., Niu, Z., Zheng, Q., Zhang, C., Ye, J., Dai, G., et al. (2018). Zn-based eutectic mixture as anolyte for hybrid redox flow batteries. *Scientific Reports 2018 8:1* 8, 1–8. doi:10.1038/s41598-018-24059-x.
